# Clinico-Pathological Profile and Outcomes of 45 Cases of Plasma Cell Gingivitis

**DOI:** 10.3390/jcm10040830

**Published:** 2021-02-18

**Authors:** Stefania Leuci, Noemi Coppola, Nicola Adamo, Maria Eleonora Bizzoca, Daniela Russo, Gianrico Spagnuolo, Lorenzo Lo Muzio, Michele Davide Mignogna

**Affiliations:** 1 Department of Neurosciences, Reproductive and Odontostomatological Sciences, Oral Medicine Unit, University of Naples Federico II, 80131 Naples, Italy; stefania.leuci@unina.it (S.L.); Nicola.adamo@live.com (N.A.); gspagnuo@unina.it (G.S.); mignogna@unina.it (M.D.M.); 2 Department of Clinical and Experimental Medicine, University of Foggia, 71122 Foggia, Italy; marielebizzoca@gmail.com (M.E.B.); lorenzo.lomuzio@unifg.it (L.L.M.); 3 Department of Advanced Biomedical Sciences, Pathology Section, University of Naples Federico II, 80131 Naples, Italy; danielarusso83@yahoo.it

**Keywords:** plasma cell gingivitis, plasma cell mucositis, inflammation, gingivitis, plasma cell

## Abstract

Plasma cell gingivitis (PCG) is an infrequent inflammatory disease of the gingiva of unknown etiology, characterized by a dense polyclonal proliferation of plasma cells in the connective tissue. The aim of this study was to present a case series of patients affected by PCG, analyzing demographic, clinical, histopathological, and therapeutic data. A group of 36 females and 9 males with a mean age of 60.3 years was evaluated. Clinically, 25 cases were bullous, a clinical phenotype never reported to date, 4 erythematous, 4 keratotic, 4 verruciform, and 3 ulcerative. On histological examination, pure polyclonal plasma cell infiltrate was detected in 20 specimens, while in 25 specimens it was associated with a mixed infiltrate. The first-line therapy consisted of oral hygiene and topical corticosteroids in all patients. In 25 patients, doxycycline and sulfasalazine were added; in 10 of these patients, the disease persisted, and it was necessary to resort to systemic steroids. This study presented the clinico-pathological profile and outcomes of a case series of PCG. This could be an aid for clinicians to be aware of the heterogeneous clinical phenotype and of the possible pure bullous phenotype of PCG. Further studies are needed to improve the knowledge about this disorder.

## 1. Introduction

Plasma cell gingivitis (PCG) is a rare inflammatory benign oral condition typified by polyclonal plasma cell proliferation in the lamina propria of the oral mucosa, specifically in gingival epithelium [1,2]. PCG is also known as allergic, atypical, or idiopathic gingivostomatitis, and plasmacytosis [2,3]. Non-neoplastic proliferative plasma cell infiltration can involve different anatomical sites, included the upper aerodigestive tract, and it is broadly characterized by heterogeneous morphological features, high morbidity, and a lack of shared knowledge about the therapeutic approach [4,5]. From the few data in the literature, the average age of onset is 45 years and there is a higher prevalence in males [1]. Although etiology is still unclear, many hypotheses have been postulated regarding its onset, ranging from hypersensitive reactions to certain types of antigens (e.g., chewing gum components [6,7,8,9,10], toothpaste [4,11], khat [12,13,14,15], or specific foods [16,17,18]) to a physical somatization of psychological disorders [19]. However, the majority of these lesions are considered to be idiopathic [1]. Pathogenetically speaking, chronic inflammatory modifications could lead to loco-regional immunological dysregulation and could induce plasma cell migration with pro-inflammatory cytokines that seem to have a key role in the immune-mediated mechanism that triggers B-cell proliferation [20].

Clinically, PCG occurs in the majority of cases as painful, edematous, and/or erythematous lesions, with or without erosions or ulcerations which easily bleed [21,22,23,24]. The histological appearance consists of a dense and massive plasma cell infiltration into subepithelial connective tissue. PCG could mimic a wide range of life-threatening entities such as squamous cell carcinoma, autoimmune mucocutaneous bullous diseases (AMBDs), and lymphoproliferative disorders [2,25,26]. Plasma cell proliferation is also associated with some infectious diseases, such as syphilis [27], Castleman’s disease [28], a primary infectious disease of the lymph node, and, recently, COVID-19 [29]. Thus, early diagnosis is required. Based on heterogeneous clinical features and the absence of a pathognomonic histological report, diagnosis and management still represent a challenge for clinicians. Moreover, management lacks an international consensus about drug classes and regimens, often resulting in a poor clinical outcome. Treatment is usually symptom-based, with corticosteroids [22,30], immunomodulators [20,31], antibiotics [32], and plaque-control local mouthwashes that represent the most frequent choice as first-line therapy, in order to delete local irritants and to reduce immunological cell-mediated and cytokine-mediate response, preventing recovery.

The main aim of this study was to describe a retrospective case-series of patients affected by PCG, analyzing demographic characteristics, clinical features, histological findings, and management.

## 2. Materials and Methods

This was a retrospective observational study on a case series carried out in our Oral Medicine Unit, compliant with the ethical principles of the World Medical Association Declaration of Helsinki and approved by the Local Ethics Committee (N° 69/19). A medical chart review of all patients with a confirmed diagnosis of PCG was conducted by two independent oral medicine specialists between January 2000 and September 2020. The following data were collected: Demographics (age at the time of the first access to our clinic and gender), laboratory investigations (complete blood count, blood glucose levels, urine tests), clinical features (type and site), clinical diagnosis, histopathological features, final diagnosis, and treatment. Data regarding histological findings were retrieved from the electronic database of the Department of Advanced Biomedical Sciences, Federico II University of Naples.

Each patient underwent a complete intra- and extra-oral examination performed by an oral medicine specialist and a detailed assessment of full mouth plaque score (FMPS), probing depth (PD), and clinical attachment level (CAL) performed by a periodontist. Subsequentially, an incisional biopsy was made by an oral medicine specialist. A single biopsy specimen, at least 5 mm, was obtained for each patient, including lesional and perilesional area, avoiding de-epithelialized or infected areas. Each specimen was placed in 10% buffered formalin solution for fixation; paraffin-embedded material was cut into 4 μm-thick sections and stained with hematoxylin/eosin for routine histological examination using an optical microscope (Model Olympus CX41RF, Olympus Corporation, Tokyo 163-0914, Japan) at magnifications of 40× and 100× by a general pathologist. In cases with erythematous, bullous, erosive, or ulcerative phenotype, direct immune-fluorescence microscopy (DIF) was made to detect deposits of Igs-G, Igs-A, Igs-M, C3, and fibrinogen, and an ELISA test was performed to detect antibodies against Desmoglein 1 and 3, BP 180, and BP 230.

All data collected from each patient were analyzed using descriptive statistics. The qualitative variables were described by frequency, mean, and standard deviation and were calculated for quantitative variables.

## 3. Results

The demographic and clinical characteristics and therapeutic regimen of the patients are shown in the Table 1. A total of 45 Caucasian patients with a diagnosis of PCG were identified: 36 females (80%) and 9 males (20%). The mean age was 60.3 years (median = 61 years), ranging between 11 and 82 years. The involved gingival localizations were: Maxilla in 29 cases (64.4%), mandible in 6 cases (13.3%), and both in 10 cases (22.2%). In 13 cases (28.9%), the lesions also involved the alveolar mucosa.

**Figure 1:**
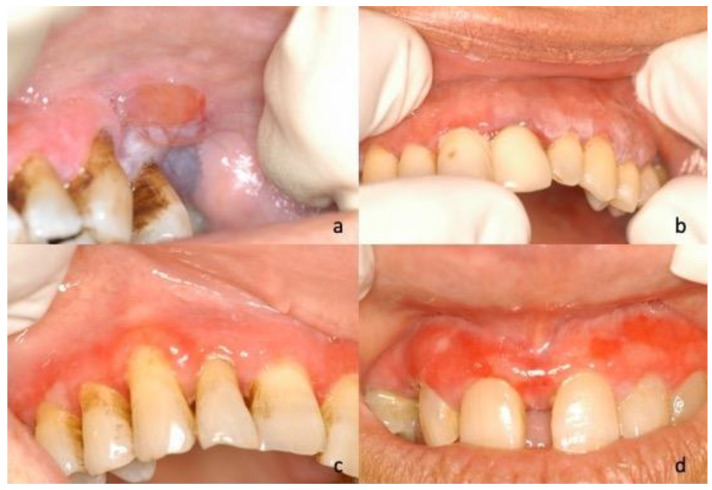
(**a**) Photograph of 51-year-old male, blistering lesion in the upper left gingival with a posterior hemorrhagic lesion; (**b**) 57-year-old female, clinical appearance of a maxillary blistering lesions in the upper left area. In both (**a**) and (**b**), the white aspect of the marginal gingiva is not related to a keratinization, but represents the partial collapsed roof of the bulla. (**c**) Photograph of 60-year-old female, ulcerative aspect of the marginal gingiva in the upper right site where it is possible to identify one intact bulla on 13 and a collapsed roof of the bulla on 15; (**d**) 74-year-old female, ulcerative aspect of the adherent gingiva in the frontal area of the maxilla where it is possible to identify mixed hypertrophic lesions.

**Figure 2:**
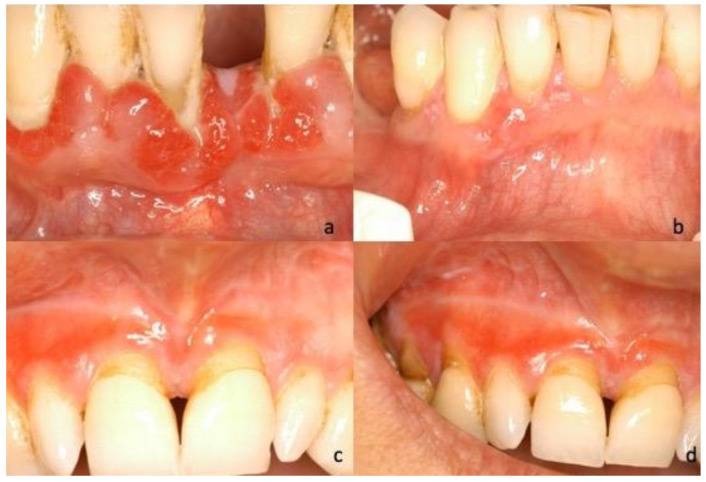
(**a**) Photograph of 76-year-old male, multiple hypertrophic lesions with erythema of the right mandible; (**b**) 81-year-old male, localized erythema of the marginal gingiva between 42–43. (**c**,**d**) Photographs pf 58-year-old female, mixed lichenoid aspects (keratinization and erythema) of the marginal gingiva and alveolar mucosa of the maxilla.

**Figure 3:**
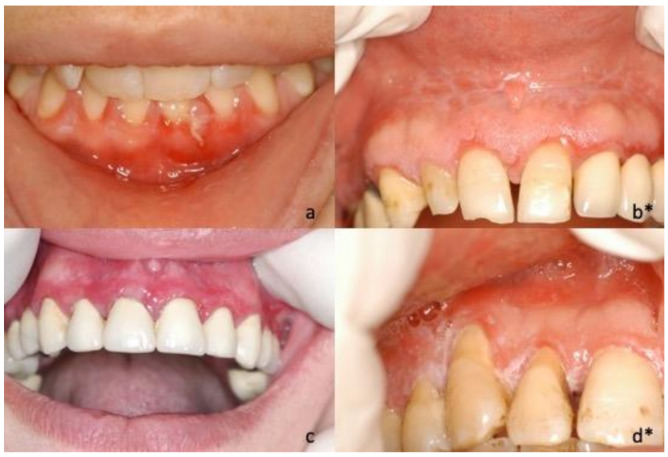
(**a**) Photograph of 36-year-old female, extensive blistering and ulcerative lesion of the marginal gingiva which extend beyond alveolar mucosa; (**b**) * 58-year-old female, reticular keratinization of the alveolar mucosa associated to homogeneous keratotic plaque of the adherent gingiva; it is possible to identify focal erythema in the marginal sites; (**c**) 56-year-old female, diffuse ulcerative and blistering lesions localized in the gingival area of the maxilla; (**d**) * 62-year-old female, focal keratinization in the papillary area of the upper right maxilla; the alveolar mucosa appears erythematous. * Clinically, four cases showed mixed lichenoid aspects which histological examination showed a dense plasma cell infiltrate, and it did not show a subepidermal band-like T CD8+ lymphocyte infiltrate.

**Figure 4:**
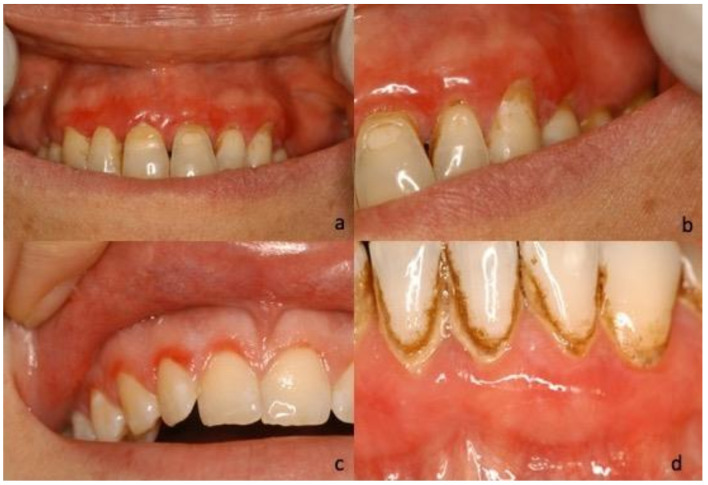
(**a**,**b**) Photograph of 67-year-old female, extensive erythematous lesion of all adherent gingiva in the maxilla; (**c**) 23-year-old female, erythema localized on the marginal site of 11, 12, 13; (**d**) 81-year-old female, erythematous lesions localized in the frontal area of the mandible.

During the first oral examination, clinicians provided the description of the lesions in 40 of the 45 cases, reporting the prevailing clinical phenotype/clinical diagnosis for each of them: Twenty-five (62.5%) were described as bullous lesions/unspecific blistering mucositis, four (10%) as erythematous lesions/erythroplakia, four (10%) as keratotic lesions/oral lichenoid lesions, four (10%) as verruciform lesions/suspected malignant lesions, and three (7.5%) as ulcerative lesions/unspecified (Figure 1, Figure 2, Figure 3 and Figure 4).

No abnormalities were found in laboratory tests performed, such as number of red blood cells and platelets, number and type of white blood cells, amount of hemoglobin, or urinary tests.

**Figure 5:**
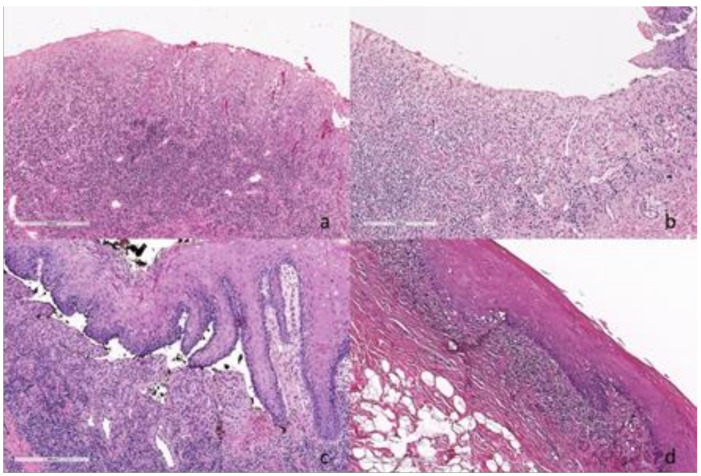
Histopathological examination with hematoxylin and eosin staining, original magnification 10×; the slides were digitized with an Aperio AT2 scanner with 20× optics. (**a**) Histopathological photographs of plasma cell gingivitis (PCG) with mixed infiltrate. (**b**) Histopathological photographs of PCG with predominant plasma cell infiltrate. (**c**) Histopathological examination of a bullous case of PCG. (**d**) Histopathological examination of PCG with lichenoid-like aspects.

Histological examination of all samples (N = 45) showed a dense epithelial and sub-epithelial infiltrate with a predominant plasma cell component, the formation of lymphoid follicular structures, and the presence of Russell bodies. In particular, pure plasma cell infiltrate was detected in 20 (44.4%) specimens, while in 25 (55.6%) specimens, although prevalent, it was associated with a mixed infiltrate with the following frequency: 17 (37.8%) with lymphomonocytes, 5 (11.1%) with eosinophils, and 3 (6.7%) with granulocytes (Figure 5). Dysplasia was detected in 9 (20%) cases; 4 (8.9%) referred to as mild, and 5 (11.1%) as moderate. Immunohistochemistry showed no restrictions for light or heavy chains in any of the analyzed samples. DIF was made in 32 doubtful cases, of which 25 were bullous, 4 erythematous, and 3 erosive/ulcerative lesions. In 4 specimens, DIF resulted positive with Ig-A and Ig-G deposits in the stromal area (2 cases) and intercellular Ig-G deposits (2 cases). In these 4 cases, ELISA tests against desmogleins 1 and 3, BP180, BP230, and IMMUNOBLOTTING were negative.

All patients started firstly plaque-control mouthwashes (chlorhexidine solution 0.20%, twice a day) with dental hygiene in association to topical steroids. Topical treatments consisted of betamethasone, 2 mg diluted in 5 mL of saline solution used as mouthwashes twice or three times a day, and/or clobetasol propionate applied with gingival trays twice a day. In 22 (48.9%) cases we also used topical pimecrolimus applied twice a day with trays. The duration of topical treatments ranged from 2 to 6 weeks. In 25 (55.6%) patients, this therapeutic protocol has been implemented with sulfasalazine, 500 mg, and doxycycline, 100 mg, twice a day for 14 days, achieving a better response to treatment. In 10 (22.2%) of these patients, the disease persisted, and it was necessary to resort to systemic steroids with betamethasone, 4 mg/day, or prednisone, 50 mg/day (tapered by 5 mg every other day). However, in many cases (11.1%), steroids failed to guarantee therapeutic success over time and disease relapse occurred when their use was stopped or when the dose was decreased. Therefore, these cases were treated with systemic immunosuppressants and/or immunomodulators, such as colchicine, mycophenolate mofetil, 750 mg twice daily, and azathioprine, 100 mg daily.

As shown in Table 1, to regard a possible correlation between clinical phenotype, histological findings, and the response to therapy, it can be supposed that cases with clinical bullous phenotype are less responsive to therapy than the other clinical phenotypes, and cases with mixed infiltrate are more responsive to therapy than in cases with pure plasma cell infiltrate.

## 4. Discussion

PCG and its extensive form plasma cell mucositis (PCM) still represents an uncommon disease with a lack of information about etiology, clinical aspects, and management. Clinician’s decisions greatly depend on clinical and histopathological data reported on few published case reports/case series. We presented a large single case series of 45 new diagnosed patients with PCG with detailed clinical and histopathological features, retrospectively collected in our Oral Medicine Unit from 2000.

Demographics showed 60.3 years as the mean age with a range of 11–82 years, which was quite different from published reports which described a lower value equal to 45 years and a much higher prevalence in women compared to men (4:1), unlike what was reported by Solomon, which showed a slight male predominance [1]. As reported in literature, gingiva is the most frequent site in patients affected by PCM; hence, we selected only the cases with gingival involvement and/or extension beyond mucus gingival junction, and in about 30% of patients the alveolar mucosa was also involved [23].

According to our anamnestic data, the etiology unfortunately still remains unknown. Only in a few patients was it possible to identify a specific local irritant, and a proven clinical remission that follows its removal, usually aided with non-surgical periodontal treatment [12,33]. Sporadic speculations were formulated about the possible roles that *Candida albicans* [6,10,34], herpes virus [35], and chronic mechanical damage [25,36,37] may or may not have played in the pathogenetic mechanism. Several cases of plasma cell lesions have been documented in “plaque-related” anatomical areas, associated with generalized periodontitis [12,33], but the role of dental plaque in PCG is still confusing and worthy of further studies [38,39]. The presence of a plasma cell infiltrate can be found in plaque-related gingival hyperplasia and chronic periodontitis, which therefore fall within the differential diagnosis [40]. The periodontal inflammatory indices of our patients did not report significant data. Alveolar bone loss, described in a previously published PCG case [41], was not detected radiographically. These data allowed us to exclude periodontal disease. Obvious structural alterations of hard and soft tissues could therefore have an immune-mediated genesis different from periodontal disease. Gingival lesions solely plaque-related regress after periodontal therapy, unlike what occurs in PCG. The exact role of plaque in the onset of PCG is still unclear [38,39]. Moreover, since PCG affects the full width of the gingival epithelium it differs from plaque induced gingivitis that affects mainly marginal epithelium. The topography of the lesions, combined with the absence of necrotizing ulcerative lesions with rapidly evolving and the absence of decapitated papillae, allows for a differential diagnosis with the acute necrotizing ulcerative gingivitis in cases of PCG with ulcerative phenotype.

Clinically, our cohort of patients showed lesions with erythematous, erosive/ulcerative/fissuring, and keratotic and verruciform aspects, mimicking oral squamous cell carcinoma (OSCC), or mixed lichenoid forms, confirming the clinical polymorphism of the disease. Moreover, for the first time, we described a pure bullous phenotype in 25 patients, of which there has been no evidence so far in the literature. Clinical diagnosis was confirmed by positive Nikolsy’s sign, indicating only the split of the epithelium through the lifting of the bulla’ roof. A gingival blister can resemble any bullous mucositis, and this is why differential diagnosis includes iatrogenic/traumatic mucositis, autoimmune blistering diseases, interface lichenoid mucositis, and erythema multiforme [42]. For a proper diagnosis, it is necessary to complete the diagnostic algorithm with histological and immunological data on the tissue (DIF) and in the sera (ELISA, immunoblotting). Histological examination of incisional biopsy stained by hematoxylin-eosin, the first step of the diagnostic workflow, shows the level of the bulla, intraepithelial or subepithelial, and the type of inflammatory infiltrate [43] followed by the DIF and ELISA test for desmoglein 1 and 3, BP180, and 230 [44]. In our bullous, but also in erythematous/erosive cases, histological analysis showed only subepithelial detachment; DIF resulted positive for Igs-A and Igs-G in the stromal area and intercellular deposits of Igs-G in 4 specimens. In all the blistering types of our group, ELISA and immunoblotting tests for the major antigens commercially available were negative, allowing us to rule out the diagnosis of AMBDs.

In the remaining 20 cases, histological data revealed the presence of stratified, squamous, in some cases parakeratinized, in some cases hyperplastic, epithelium overlying a densely infiltrated connective tissue. There was a dense infiltrate of plasma cells in 44.4% of the 45 examined specimens, and in the remaining cases, the inflammatory infiltrate was mixed; however, the plasma cell component was always predominant. Plasma cell infiltrate was found predominantly in the chorion and only in some cases in the perivascular site. In a minority of cases (5 cases), it had a “band-like” arrangement at the dermal epidermal junction mimicking a lichenoid pattern. These cases were difficult to clearly identify as plasma cell gingivitis. However, plasma cells were the most represented cells and no T CD8+ lymphocytes were detected in the lichenoid-like arrangement; therefore, the diagnosis of plasma cell gingivitis was made. In all specimens, a high CD38 expression was observed on plasma cells. In many cases, histological analysis also highlighted process of neovascularization in the stroma associated with chronic inflammation which occasionally may have a peri-glandular arrangement. In some ulcerative forms, we encountered inflammatory granulation tissue with abundant eosinophil infiltration and actinomyces colonization, which, however, did not appear to have a pathogenetic role in this clinical manifestation.

Moreover, histological analysis showed dysplasia in 9 cases, 5 of which reported as moderate grade. There were no data on the risk of oncological transformation arising on PMC; there is only one documented case of a neoplastic transformation to squamous cell carcinoma [45] plus another documented case where the oral disorder was a sign of an undergoing systemic monoclonal gammopathy [46]. In the absence of reliable data, it is not possible to establish a protocol regarding oncological follow-up. Moreover, through immunohistochemistry, malignancies, such as multiple myeloma, were then ruled out in all specimens. Immunohistochemistry in PCM is usually performed to confirm Ig-s polyclonal, in the absence of obvious restrictions for light or heavy chains, and thus confirm that the lesion is a manifestation of a non-neoplastic polyclonal benign reactive process [47].

Regarding drug therapy, the initial phase of the treatment consisted of oral hygiene instructions, scaling, and topical treatments with corticosteroids in all patients [30]. In recalcitrant cases, topical pimecrolimus was used [20]. In our experience, in about half of patients, to obtain good results in terms of the reduction of the number of lesions and symptoms, in association with topical treatments, sulfasalazine, an anti-inflammatory drug that inhibits TNF-alpha, and doxycycline, used as an immunomodulator and antibacterial, were used. In unresponsive cases, patients underwent systemic treatments with betamethasone or prednisone. In approximately 10% of patients, complete remission of the disease was achieved with systemic immunosuppressants and/or immunomodulators, such as colchicine, mycophenolate mofetil [2], and azathioprine [48]. In our cohort, the tendency to relapse was low and no maintenance therapy was used.

The physician that deals for the first time with PCG faces a remarkable firstly diagnostic and secondly therapeutic dilemma. This is due to the poor clinical history characterization of the disorder that, in turn, greatly depends on the lack of knowledge relative to etiology and pathophysiological mechanisms. The most important step in the diagnostic pathway is to recognize and consequently to exclude systemic plasma cell disorders: Monoclonal gammopathy of undetermined significance, multiple myeloma, lymphoplasmacytic lymphoma/Waldenstrom macroglobulinemia, amyloidosis, and POEMS syndrome (polyneuropathy, organomegaly, endocrinopathy, monoclonal protein, and skin changes) [49], some of which, such as amyloidosis, plasmacytoma of bone, extramedullary plasmacytoma, and multiple myeloma, can show osseous and extra-osseous growth patterns in the head and neck [50].

All of these disorders are characterized by the detection of a monoclonal paraprotein in the serum or urine and/or the presence of monoclonal plasma cells in the bone marrow space, or, rarely, in other tissues. In this context our algorithm encompassed: (a) Physical exam and history, (b) complete blood count (number of red blood cells and platelets, number and type of white blood cells), (c) amount of hemoglobin, (d) blood chemistry studies and serum protein electrophoresis, (e) blood and urine immunoglobulin studies for free light chain quantification (i.e., for multiple myeloma, beta-2-microglobulin, M protein, free light chains), (f) twenty-four-hour urine test, and (g) immunochemistry on oral mucosa specimen to evaluate the plasma cells mono/polyclonality.

Plasma cell infiltrate can be found in other systemic diseases such as Castleman’s disease which rarely involves the head and neck region. Affected patients usually show isolated or multiple lymphadenopathies, and in the multicentric type it is associated with systemic symptoms such as fever, anemia, and hepatosplenomegaly [51]. In our cases, neither signs and/or symptoms were present.

Clinicians should also be aware of the possible pure bullous phenotype of PCG in order to take into consideration this possible and less grievous differential diagnosis if compared to other blistering oral diseases. More research should be carried out in order to further our knowledge of this disease.

## Figures and Tables

**Table 1:** Demographic, clinical, and histopathological characteristic and therapeutic regimen.

N°	Sex	Age	Clinical Phenotype	Dysplasia	Infiltrate	Treatment			
						First-Line *	SFZ/DOX	Systemic Steroids	Immunosuppressants *^1^
1	F	72	Bullous	No	M-E	X	X		
2	F	76	Bullous	No	PP	X	X		
3	M	56	-	No	PP	X			
4	F	66	Bullous	No	PP	X			
5	F	36	Bullous	Yes	PP	X	X	X	
6	F	56	Bullous	No	PP	X	X	X	X
7	F	59	Bullous	No	PP	X	X	X	
8	F	45	Bullous	Yes	M-G	X	X		
9	F	58	Bullous	No	PP	X	X		
10	F	62	Bullous	No	PP	X			
11	F	53	Bullous	No	M-L	X	X	X	X
12	M	51	Bullous	No	M-L	X	X		
13	F	62	Bullous	No	PP	X	X	X	
14	F	72	Bullous	No	PP	X			
15	F	57	Bullous	No	M-L	X	X	X	
16	M	52	-	No	M-L	X			
17	M	58	Bullous	No	M-E	X	X	X	X
18	F	68	Bullous	No	M-L	X			
19	M	79	Bullous	No	M-E	X			
20	F	61	Bullous	Yes	PP	X	X		
21	F	48	Bullous	No	M-E	X	X	X	X
22	F	68	Bullous	No	PP	X			
23	F	67	Bullous	No	PP	X	X	X	X
24	M	79	-	No	M-L	X			
25	M	63	Bullous	No	M-L	X	X		
26	F	56	Bullous	Yes	PP	X	X	X	
27	F	53	Bullous	No	M-L	X	X		
28	F	64	Bullous	No	M-L	X	X		
29	F	60	Ulcerative	No	M-L	X	X		
30	F	58	Keratotic	Yes	PP	X			
31	F	62	Keratotic	No	PP	X			
32	F	74	Ulcerative	No	PP	X	X		
33	F	58	Keratotic	Yes	M-L	X	X		
34	F	72	Keratotic	No	M-L	X			
35	F	81	Erythematous	Yes	M-G	X			
36	F	11	-	No	M-L	X			
37	F	37	-	No	M-L	X			
38	F	75	Verruciform	No	PP	X			
39	F	82	Ulcerative	No	M-L	X			
40	M	81	Erythematous	No	PP	X			
41	F	54	Verruciform	No	PP	X			
42	F	23	Erythematous	No	M-L	X	X		
43	M	76	Verruciform	Yes	M-L	X			
44	F	67	Erythematous	Yes	M-G	X	X		
45	F	44	Verruciform	No	M-E	X	X		

M-E: Mixed infiltrate with eosinophils; PP: Pure plasma cell infiltrate; M-G: Mixed infiltrate with granulocytes; M-L: Mixed infiltrate with lymphomonocytes; *: Plaque control + chlorhexidine solution 0.20% + topical steroids; SFZ: Sulfasalazine; DOX: Doxycycline; *^1^: Azathioprine or colchicine or mycophenolate mofetil.

## Data Availability

The data that support the findings of this study are available from the corresponding author upon reasonable request.
